# PyDISH: database and analysis tools for heme porphyrin distortion in heme proteins

**DOI:** 10.1093/database/baaa066

**Published:** 2020-10-03

**Authors:** Hiroko X Kondo, Yusuke Kanematsu, Gen Masumoto, Yu Takano

**Affiliations:** School of Regional Innovation and Social Design Engineering, Faculty of Engineering, Kitami Institute of Technology, 165 Koen-cho, Kitami, Hokkaido 090-8507, Japan; Department of Biomedical Information Sciences, Graduate School of Information Sciences, Hiroshima City University, 3-4-1 Ozukahigashi Asaminamiku, Hiroshima 731-3194, Japan; Laboratory for Computational Molecular Design, RIKEN Center for Biosystems Dynamics Research, 6-2-3, Furuedai, Suita 565-0874, Japan and; Department of Biomedical Information Sciences, Graduate School of Information Sciences, Hiroshima City University, 3-4-1 Ozukahigashi Asaminamiku, Hiroshima 731-3194, Japan; Information Systems Division, RIKEN Head Office for Information Systems and Cybersecurity, 2-1 Hirosawa, Wako, Saitama 351-0198, Japan; Department of Biomedical Information Sciences, Graduate School of Information Sciences, Hiroshima City University, 3-4-1 Ozukahigashi Asaminamiku, Hiroshima 731-3194, Japan

## Abstract

Heme participates in a wide range of biological functions such as oxygen transport, electron transport, oxygen reduction, transcriptional regulation and so on. While the mechanism of each function has been investigated for many heme proteins, the origin of the diversity of the heme functions is still unclear and a crucial scientific issue. We have constructed a database of heme proteins, named **Py**thon-based database and analyzer for **DIS**tortion of **H**eme porphyrin (PyDISH), which also contains some analysis tools. The aim of PyDISH is to integrate the information on the structures of hemes and heme proteins and the functions of heme proteins. This database will provide the structure–function relationships focusing on heme porphyrin distortion and lead to the elucidation of the origin of the functional diversity of heme proteins. In addition, the insights obtained from the database can be used for the design of protein function. PyDISH contains the structural data of more than 13 000 hemes extracted from the Protein Data Bank, including heme porphyrin distortion, axial ligands coordinating to the heme and the orientation of the propionate sidechains of heme. PyDISH also has information about the protein domains, including Uniprot ID, protein fold by CATH ID, organism, coordination distance and so on. The analytical tools implemented in PyDISH allow users to not only browse and download the data but also analyze the structures of heme porphyrin by using the analytical tools implemented in PyDISH. PyDISH users will be able to utilize the obtained results for the design of protein function.

**Database URL**: http://pydish.bio.info.hiroshima-cu.ac.jp/

## Introduction

**Figure 1.**
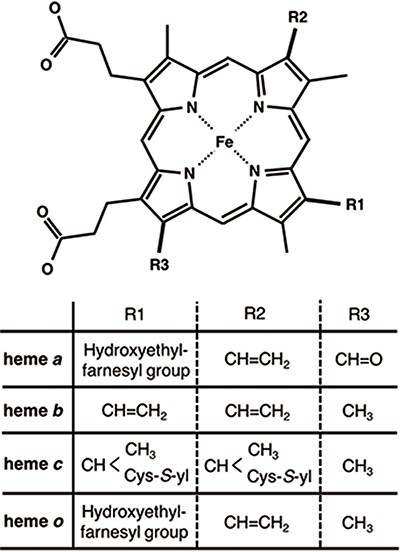
Chemical structures of hemes. R1, R2 and R3 in the upper structure correspond to those in the lower table. Heme *c* forms covalent bonds between the ethenyl groups in heme and the thiol groups of Cys residues in its host protein.

Heme proteins are a group of proteins containing one or more hemes as a cofactor and play diverse and important roles in biological functions, including oxygen transport/storage, electron transfer, redox reactions, transcriptional regulation (1–4) and so on. Scientists in biomedicine and white biotechnology are keenly interested in the structural prediction and design of novel heme proteins (5–7). Heme, the active site of heme proteins, consists of an iron ion coordinated to a porphyrin. Whereas the structures and functions of many heme proteins have been characterized, the origin of the functional diversity of heme remains unclear. To address this crucial scientific issue, comprehensive investigations of the hemes in heme proteins are required. Some factors may regulate the chemical properties of hemes. Heme is classified into various types on the basis of the peripheral groups, as shown in Figure 1. Heme *c* forms covalent bonds with its host proteins, while hemes *a, b* and *o* bind them noncovalently. The most common heme types among the known heme proteins are hemes *b* and *c* (8). The axial ligands coordinating to the heme iron ion can also be attributed to the electronic and spin states of hemes. In addition to the effects of the axial ligands and/or types of heme on the chemical properties of heme proteins, heme distortion has recently been suggested to control protein functions (9–14). Therefore, the elucidation of the structure–function relationships of heme is important for clarifying the functional mechanisms of heme proteins and designing novel heme proteins. Due to the relentless pursuit of protein tertiary structure determinations, since the earliest crystallographic studies on myoglobin and hemoglobin (15, 16), an abundance of heme protein structural data is now available in the Protein Data Bank (PDB) (17). More than 5000 heme protein PDB entries are registered, as of March 2020. Considering the important role of comprehensive investigations from the viewpoint of structure–function relationships, toward a deeper understanding of the origin of the various functions of heme proteins, the development of a heme protein database with a platform for statistical analysis is a fundamental issue in structural biology.

 The PDB is the only global public data bank for experimentally obtained 3D-structures of proteins and nucleic acids. Although users can access all published data, for people unfamiliar with computational analysis or bioinformatics, it is time-consuming to cyclopedically analyze these structural data. Consequently, secondary databases with sub-datasets from the PDB have been developed (18–23). Secondary databases for metalloproteins were also constructed. The PROMISE database (24) was a database for bioinorganic motifs in proteins and focused on the relationships between polypeptides and active sites including chlorophylls, binuclear iron centers, hemes, iron-sulfur clusters, molybdopterins and mononuclear iron centers. Though PROMISE was a useful information source, it is now unavailable. The Metalloprotein Database and Browser (MDB) (25) provided structural information on protein metal-binding sites and was utilized to obtain their geometrical information, such as the coordination distances and angles, but it is also currently unavailable. The Heme Protein Database (HPD) (8), which provides the information on protein folds, heme types, axial ligands and reduction potentials of heme proteins, is even now available. HPD is an excellent resource for the investigations of the structure–function relationships in heme proteins. However, it is not useful for structural analyses of heme porphyrins and has probably not been updated since it was released in 2008.

**Figure 2.**
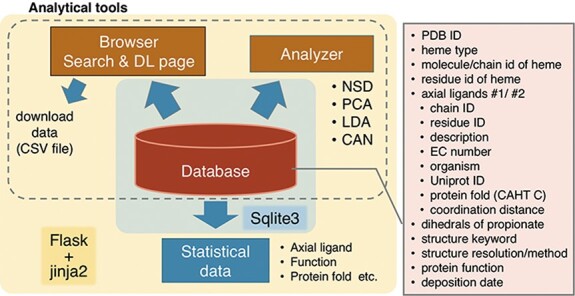
Overview of PyDISH.

We are in the process of developing a database, **Py**thon-based database and analyzer for **DIS**tortion of **H**eme porphyrin (PyDISH). In constructing PyDISH, we focus on the distortions of heme porphyrin as well as heme types and axial ligands and emphasize on the analysis using the data in PyDISH by users themselves. We have been collecting the following heme protein data: porphyrin distortions, axial ligands, coordination distances, orientations of propionate sidechains, protein functions, resolutions of structural data and protein folds from the PDB, Uniprot (26) and CATH database (20, 27). Although we believe that the information about the protein environment other than the axial ligands of hemes is also important to clarify the structure–function relationships in heme proteins, considering the previous study (28), it is difficult to quantify and store the interactions between hemes and protein domains in a database, due to the diversity of hemes and requirements for the spatial information on heme-binding sites. Therefore, the present version of PyDISH only includes the coordination distances of the axial ligands. The statistics of amino acid residues in heme proteins and the differences in the tertiary structures between apo- and holo-proteins were analyzed in previous studies (29, 30). PyDISH has the advantage of providing ‘a non-datascientist-friendly platform for the statistical analysis’. Statistical data for a set of nonredundant heme proteins are also available. The insights obtained by analyses with PyDISH will lead to the functional predictions of unknown heme proteins and the *de novo* design of protein functions.

## Database architecture

### Overview and implementation of PyDISH

An overview of PyDISH is shown in Figure 2. In PyDISH, an entry is created for one heme molecule in a PDB entry, with information about the axial ligands and the protein domains including them, as listed in the right panel of Figure 2. For the specification of each heme, each PyDISH entry has the chain ID, molecule ID (asym ID) and residue index of the heme in the PDB entry, in addition to the PDB ID. The atomic coordinates of the Fe-porphyrins of hemes and evaluations of their distortions are also available. To facilitate the use of these data, we implemented ‘Browser’ and ‘Analyzer’ in PyDISH. Users can select and browse the dataset in PyDISH by using Browser and can easily perform the structural analysis of heme porphyrins by using Analyzer. The data extracted in Browser and the results obtained in Analyzer can be downloaded for users’ own analyses. The statistical data of axial ligands, protein functions, etc., are also available, for the whole data stored in PyDISH (‘Data -> Data in PyDISH’ page) and the nonredundant datasets (‘Data -> Statistical data’ page).

 The PyDISH web interface is written in HTML and JavaScript and uses Cascading Style Sheets (CSS) at the front end. As shown in Figure 2, SQLite is used to store and query the dataset, and Flask and jinja2 are used as the web application framework and the HTML templating engine, respectively. For the implementation of analysis tools in Analyzer, the scikit-learn (31), MDTraj (32) and Matplotlib (33) Python libraries and Jmol: an open-source Java viewer for chemical structures in 3D (http://www.jmol.org/) are used. In the following subsections, we describe the details of the stored data and introduce the Browser and Analyzer.

### Data collection

**Figure 3.**
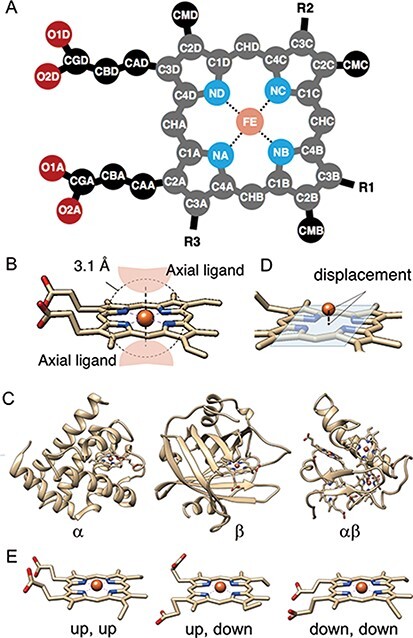
(A) The atomic nomenclature in heme. Twenty-five atoms of Fe-porphyrin (shown in lighter colors: grey, cyan and orange) represent the porphyrin skeleton. (B) Identification of the axial ligand(s) of heme. The amino acids or other small molecules within 3.1 Å of the FE atom were identified as the axial ligands. (C) Representative structures of each protein fold in CATH level C (1: α, 2: β and 3: αβ). (D) The out-of-plane displacement of the iron ion from the least square fit plane of the 4 nitrogen atoms (NA, NB, NC and ND). (E) Schematic diagrams of the orientations of the propionate sidechains.

The tertiary structures (mmCIF formatted files) of heme proteins were extracted from the PDB. We searched for proteins with the compound ID of HEM, HEA, HEB, HEC or HEO, by using the SQL search in PDBj Mine (34) of PDBj (35) (https://pdbj.org/mine). In total, 5259 unique entries were extracted from 158 180 entries as of 30 November 2019. From each entry, we extracted hemes with intact data of the atomic coordinates of the iron and porphyrin ring (25 atoms shown in light colors in Figure 3A) and created an entry for each heme. We used the Biopython library (36) as a parser and extracted the atomic coordinates, entity ID, chain ID and molecule ID (asym ID) of each atom from the ‘_atom_site’ data. In the case where more than two records exist for one atom, only the first one (‘PDB model number of 1 and atom site with the alternative ID of “.”’ or ‘atom site with the alternative ID of A’) was used. For the identification of each heme, each entry has a unique molecule id (asym id), a residue index and a chain ID of a heme in a PDB entry. In addition, the following information was collected for each entry: residue name of the subjected heme (HEA, HEB, HEC, HEM or HEO), axial ligands, number of axial ligands (coordination number, 0 to 2), information on each axial ligand (coordination distance and Uniprot ID, protein fold by CATH, Enzyme Commission (EC) number, organism of the protein domain including each axial ligand), orientation of propionate sidechains, keyword(s) of the PDB entry (pdbx_keywords), manually defined protein function, resolution of the X-ray diffraction data, experimental method used to determine the structure and date of initial deposition in the PDB (yyyy-mm-dd). For the heme nomenclature, HEA and HEC represent heme *a* and heme *c*, respectively. HEB includes heme *b* and *c*. The difference between these hemes is shown in Figure 1. The axial ligands, the description of the heme porphyrin distortion and the orientation of the propionate sidechains will be described in detail in the following subsection. For protein functions, similar descriptions in the structural keywords collected from ‘_struct_keywords.text’ in its PDB entry were grouped manually. The correspondence between the function in PyDISH and the keyword in the PDB entry is shown in the PyDISH web page: http://pydish.bio.info.hiroshima-cu.ac.jp/table_function_whole/.

### Axial ligands

Axial ligands were identified as the amino acids or other small molecules within 3.1 Å of the iron atom of each heme, as shown in Figure 3B. We selected the two nearest neighbors in the cases where more than three residues and/or molecules were identified. The compound name is assigned for each axial ligand by using the ‘_entity_id’ of the chain (protein domain), including the subjected axial ligand and the items of ‘_entity.pdbx_description’. The coordination distances of each axial ligand were calculated as the minimal distance between the iron atom of the heme (FE in Figure 3A) and the atoms of the subjected axial ligands. We collected the scientific name of the organism and the EC numbers for each axial ligand by using the ‘_entity_id’ and the items ‘_entity_src_gen.pdbx_gene_src_scientific_name’ and ‘_entity.pdbx_ec’ in the mmCIF formatted file, respectively. In the cases where there is no description of ‘_entity_src_gen.pdbx_gene_src_scientific_name’, we used ‘_entity_src_nat.pdbx_organism_scientific’ as the scientific name of the organism. After collecting them, we assigned their first two words to the organism and their first three class labels to the EC number. Uniprot ID and protein fold (CATH level C, Figure 3C) were also assigned to each axial ligand, by using the summary files in SIFTS (https://www.ebi.ac.uk/pdbe/docs/sifts/quick.html) and the summary file in CATH (ftp://orengoftp.biochem.ucl.ac.uk/cath/releases/daily-release/newest). The ID assig- ned to the protein domain (chain) including each axial ligand was extracted from these files.

### Description of heme porphyrin distortion

**Figure 4.**
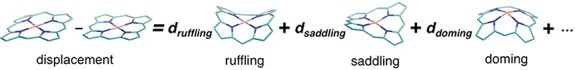
Schematic diagram of NSD. The left side represents the structural displacement from the equilibrium state. The right side is the linear combination of vibrational modes.

The distortion of heme porphyrin must be quantified to understand its effect on the chemical properties of heme. We analyzed the distortion of heme porphyrin by using normal-coordinate structural decomposition (NSD) (37). NSD is a method that represents the porphyrin distortion as displacements from its equilibrium structure with D_4h_ symmetry (a planar structure) along the normal modes. The distortion of a porphyrin ring consisting of *N* atoms can be described by the linear combination of (3*N*–6) vibrational modes as shown in Figure 4.

 Generally, a molecular structure can be expressed exactly with the distortions along all normal modes of the degrees of freedom of (3*N*–6). In the case of a planar metalloporphyrin structure with the D_4h_ symmetry, these normal modes are determined by solving the secular equation as shown in Eq (1) (38).


(1) \begin{equation*}\left| {{{\bf{M}}^{ - 1/2}}\left( {{\nabla ^2}E} \right){{\bf{M}}^{ - 1/2}} - \lambda {\bf{I}}} \right| = \left| {{1 \over {\sqrt {{m_i}{m_j}} }}{{{\partial ^2}E} \over {\partial {x_i}\partial {x_j}}} - \lambda {\delta _{ij}}} \right| = 0,\end{equation*}

where **M** and **I** are the diagonal mass matrix and the identity matrix, respectively, ${\nabla ^2}E$ represents the Hessian matrix, and *δ_ij_* is the Kronecker delta. The eigenvectors (normal modes) $\widehat {\bf{Q}}_n^{{\Gamma }}$ and the corresponding eigenvalues $\lambda _n^{{\Gamma }}$ of the *n*-th mode of symmetry Γ are obtained by diagonalization of the root-mass-weighed matrix of the second derivatives of the potential energy *E* with respect to the Cartesian coordinates. The displacements along each mode are obtained by the following equation:


(2) \begin{equation*}d_k^{{\Gamma }} = {}_{}^t\widehat {\bf{Q}}_k^{{\Gamma }}{{\bf{D}}_{{\mathrm{heme}}}},\end{equation*}

where ${{\bf{D}}_{{\mathrm{heme}}}}$ is the displacement of the heme coordinates from the equilibrium structure. The structural decomposition method can be readily applied if the displacement from the equilibrium structure and the normal mode(s) are known. More detailed description of NSD were provided previously (13, 37).

The equilibrium structure and vibration modes were obtained by geometry optimizations and frequency calculations on the Fe-porphyrin complex (all sidechains of heme were replaced by hydrogen atoms), by using the PBE0 exchange–correlation functional (39) with the 6–31G(d) basis sets (40–43). We then generated vectors containing the components of the heavy atoms and normalized them for the projection of heme structures. The obtained vectors were almost orthogonal each other, as the absolute values of the self-inner products of all vectors were greater than 0.99. Only the 12 vibrational modes described in the previous study by Bikiel *et al*. (9): saddling, ruffling, doming, waving(x), waving(y), propellering, meso-stretching, *N*-pyrrole stretching, translation(x), translation(y), breathing and rotation were used in PyDISH. These vibrational modes are also shown on the web page: http://pydish.bio.info.hiroshima-cu.ac.jp/vibration_modes/ as a movie.

### Out-of-plane displacement of iron ion of heme

The out-of-plane displacement of the iron ion of heme *d_OOP_* was calculated as the distance between FE atom and the least square fit plane of the four nitrogen atoms (NA, NB, NC and ND) (Figure 3D). The least square fit plane was computed as the first and second eigenvectors of variance-covariance matrix of Cartesian coordinates of these four nitrogen atoms, **v**_PC1_ and **v**_PC2_, and the distance between the FE atom and the least square fit plane was calculated as the inner product of the relative coordinate of FE atom and the normalized third eigenvector **v**_PC3_.


(3) \begin{align*}{d_{\rm OOP}} &= {\left( {{{\bf{r}}_{FE}} - {1 \over {\# {G_{\rm atoms}}}}\mathop \sum \limits_{i \in {G_{\rm atoms}}} {{\bf{r}}_i}} \right)^{\mathrm{T}}}{{\bf{v}}_{{\mathrm{PC}}3}},\,{\mathrm{where}}\notag\\
{G_{\rm atoms}} &= \left\{ {{\mathrm{NA}},{\mathrm{ NB}},{\mathrm{ NC}},{\mathrm{ ND}}} \right\}\end{align*}

### Analysis of heme-binding pocket

The coverage of heme was analyzed for 25 atoms composing porphyrin skeleton shown as the light colors in Figure 3A. The solvent accessible surface area (SASA) was analyzed for each atom of the heme-protein complex or isolated heme (S_complex, *i*_ and S_heme, *i*_, respectively) by using MDTraj library, and the coverage *c* was calculated as follows:


(4) \begin{equation*}c =\left( \mathop \sum \limits_{i \in {G_{\rm atoms}}} {{{S}}_{{\rm heme}, i}} - \mathop \sum \limits_{i \in {G_{\rm atoms}}} {{{S}}_{{\rm complex}, i}}\right)\Bigg/\mathop \sum \limits_{i \in {G_{\rm atoms}}}{{{S}}_{{\rm heme}, i}},\end{equation*}

where *G*_atoms_ represents the set of atoms composing the porphyrin skeleton. We used only C, N, O, S and Fe atoms for the SASA calculation.

 The volume of heme-binding pocket was also analyzed by using POVME python library (44). In this analysis whole heme structure was used for the binding pocket analysis.

### Structures of propionate sidechains

The propionate sidechains also play a role in heme proteins (45–47). In order to analyze the correlation among the orientations of propionate sidechains and other properties of the heme protein, such as the porphyrin distortion and protein function, we calculated the dihedral angles of the propionate groups at C2A and C3D of the porphyrin ring (dihedral angles of C1A-C2A-CAA-CBA and C4D-C3D-CAD-CBD in Figure 3A). These data were stored in PyDISH as dihedral1 (angles of C1A-C2A-CAA-CBA) or 2 (angles of C4D-C3D-CAD-CBD). The distributions of both dihedral angles are bimodal, with peaks around − 90º and 90º, and then we defined ‘up’ (upward) or ‘down’ (downward) as a dihedral angle less or more than 0º, respectively. The representative structures are shown in Figure 3E. The composition of the combination of orientations of each propionate sidechain is as follows: 52.3% ‘up, down’, 34.5% ‘up, up’ and 13.1% ‘down, down’.

### Statistical data

We analyzed the statistics of the axial ligands, protein functions and protein folds (CATH level C). By using PISCES (48), nonredundant datasets were extracted from the whole data in PyDISH. The thresholds for sequence similarity were specified as 25% and 40%, and 228 and 423 PDB chains were culled from 11 564 PDB chains in PyDISH, respectively. We selected the PyDISH entries including these PDB chains and analyzed the compositions of axial ligand, protein function and protein fold. These results are shown on the PyDISH web page (‘Data -> Statistical data’).

**Figure 5.**
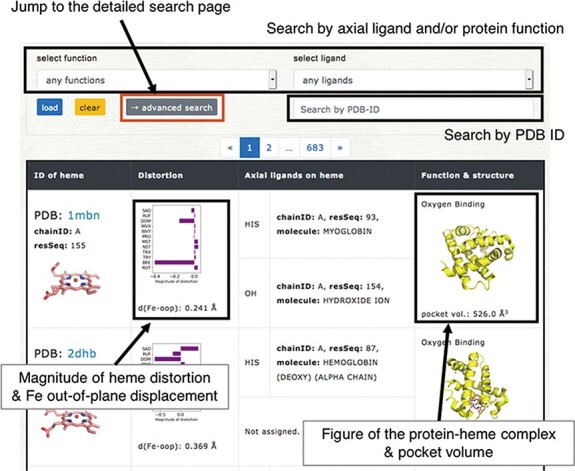
Browser in PyDISH. Select the axial ligand and protein function or enter a PDB ID, and click the ‘load’ button, and then the relevant data will be listed. The images can be enlarged by hovering the mouse cursor over each item.

The statistical data for the redundant dataset (whole data stored in PyDISH) are also shown on the web page (‘Data -> Data in PyDISH’). The major components (> 10%) are HIS-HIS (6-coordination, both upper and lower ligands are His, 23.2%), CYS (5-coordination, 13.5%) and HIS (12.1%) for the axial ligands, Oxidoreductase (55.6%), Electron transport (16.2%) and Oxygen binding (16.1%) for the protein functions, and α (78.3%) and αβ (17.0%) for the protein folds. We also analyzed the statistics of bond lengths and bond angles for the heavy atoms in the Fe-porphyrin (25 atoms) for the entries with the resolution ≤3.0 Å. Pairs of atoms within 2.2 Å of each other were defined as ‘bonded’. The bond lengths and bond angles were calculated as the distance between bonded atoms and the angle between a pair of bonded atoms with a common atom (such as C1A-CHA and CHA-C4D), respectively. There were 32 pairs of bonded atoms, and their distributions for the whole (redundant) data are also shown on the PyDISH web page (‘Data - > Structural parameters’). Almost all of the bonds lengths or angles showed a unimodal distribution, except for the bond length between C2D and C3D. The C2D-C3D length is represented by a bimodal distribution, and there is no correlation between the bond lengths and the structure resolutions

## Browser & data download

Users can browse the data in PyDISH by using ‘Browser’. In Browser, each entry is listed with the figures of the subjected heme and the complex of heme and the protein domain including its axial ligands, as shown in Figure 5. The protein function, axial ligands, description of the protein domain including each axial ligand, and the distortion of heme porphyrin (NSD), the out-of-plane displacement of FE atom, and the volume of heme-binding pocket are also listed here. The heme in each entry can be identified by the PDB ID and the asym ID (molecule ID in each PDB entry). Users can jump to each PDB entry page in PDBj by clicking on the PDB ID. The figures of hemes and heme-protein complexes were drawn with the PyMOL software (49). The entries to be displayed can be specified by the axial ligand and/or protein function or PDB ID (a regular expression cannot be used). More flexible specification by the axial ligand, the protein function and the heme type (residue name of heme) is available by using the ‘Detailed search’ page. In addition to Browser, PyDISH has the ‘Data search & download’ page (Data - > Search & download data). On this page, the list of heme proteins can be downloaded as a CSV formatted file. The dataset can be extracted by selecting the axial ligand, protein function (multiple selection enabled), EC number and organism of the axial ligand #1, maximum structure resolution (threshold) and magnitude of heme distortions (saddling, ruffling, doming and breathing). The items to be included in the results list can be also be specified. The results will be displayed in the web browser and can be sorted by the value of each item.

## Analyzer

PyDISH provides four analytical tools for the insights into heme distortion, as shown in Figure 2. One is the NSD (37), which is used to analyze the relationships between the heme distortions and axial ligand, protein function or protein fold. Two other tools are Principal Component Analysis (PCA) (50) and Linear Discriminant Analysis (LDA) (51), for the extraction of the feature vectors involved in their protein functions (chemical properties) or axial ligands. PCA is one of the most popular statistical approaches to extract uncorrelated variables that are responsible for data variances. LDA is a popular supervised learning method to extract a feature vector separating the data into two response groups. The last is Cluster Analysis with NSD (CAN). CAN is utilized to classify the porphyrin structures by their distortion (NSD). The details of these methods will be described below.

### NSD: Normal-coordinate structural decomposition

**Figure 6.**
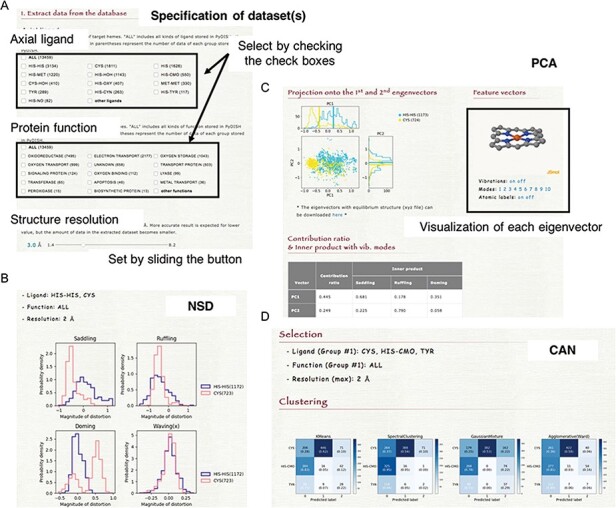
(A) How to specify the dataset(s). Select the axial ligand and the protein function according to the check boxes, and set the threshold of structure resolution by sliding the button (1.4 ~ 8.2 Å). (B) Example of the NSD result page. (C) Example of the PCA result page. The motions of the first to 10th eigenvectors can be visualized by clicking the number of modes. The contribution ratio and inner product with the saddling, ruffling and doming modes of each eigenvector are listed as a table. (D) Example of the CAN result page.

NSD is a typical approach to evaluate the heme distortions that are often associated with the chemical activity of heme. On the NSD page, the distributions of heme porphyrin distortions can be plotted per axial ligand, protein function, or protein fold (CATH level C). Users can specify a dataset of hemes for the analysis by the types of axial ligand, protein function and structure resolution, as shown in Figure 6A. The target for which the histogram is compared can also be selected. Graphs are provided for 12 vibrational modes (Figure 6B). The details of the vibrational modes used in PyDISH are described in the above section: ‘Description of heme porphyrin distortion’.

### PCA: principal component analysis

PCA is an unsupervised learning method for the dimensional reduction and provides an orthonormal basis with vectors that are uncorrelated with each other. In PyDISH atomic coordinates (Cartesian coordinates) are used as variables. Users can specify a dataset of hemes for the analysis by the types of the axial ligand, the protein function and the structure resolution and select the atoms used for the least squares fitting and those used for the PCA. After the least squares fitting of the selected heme structures by the selected atoms for the fitting to the mean coordinates, feature vectors will be calculated for the selected atoms for the PCA. The projection of atomic coordinates onto the PC1-PC2 (the first and second eigenvectors) plane and the vibrational modes along each eigenvector will be shown on the result page, as in Figure 6C. Each group in the target selected by users (axial ligand, protein function, or protein fold) will be plotted in a different color. The calculated eigenvectors (feature vectors) and the mean coordinates can also be downloaded on the result page, as an xyz formatted file.

### LDA: linear discriminant analysis

LDA is a classifier with a linear decision boundary, generated by fitting class conditional densities to the data and using Bayes’ rule. This classifier has the advantages that it has no hyperparameters to tune and does not require many computing resources. The difference from PCA is a supervised or unsupervised learning method. In PyDISH users can apply LDA to the classification of heme porphyrin structures and obtain the feature vector separating the selected two groups of hemes.

Instead of the atomic coordinates, the projected values of heme porphyrin structures onto the 12 vibrational modes of NSD are used as variables. Users specify two datasets of hemes for classification by the axial ligand, protein function and structure resolution as well as the other analysis tools, and then a feature vector classifying these datasets into two groups will be calculated. The feature vector is obtained as a linear combination of the vectors of 12 vibrational modes and can be visualized as a movie on the result page. The projections of the coordinates of hemes onto the feature vector are also plotted. The coordinates of the average structure and the feature vector can be downloaded as an xyz formatted file.

### CAN: cluster analysis with NSD

Clustering groups a set of data objects into clusters, such that the data within each cluster are more similar to one another than to those in the other clusters. All clustering methods are based on the distance or similarity between data and clusters, and each datum always belongs to one cluster. In PyDISH we introduce four clustering methods: K-means (52–54), Spectral clustering (55), Agglomerative clustering (56) and Gaussian mixture (51). The details of each method will be described below. We set the parameter value of the number of clusters as the number of target groups (axial ligand or protein function) for all methods. The other parameters of each method and its values in PyDISH are described in Table S1. The process for specifying a dataset of hemes is the same as that in NSD. The target for which the clustering is performed should be selected (axial ligand or protein function). As a result, the heat maps will be obtained for each method (Figure 6D).

K-means

K-means partitions the data into the groups of equal variances while minimizing the within-cluster variances.

Spectral clustering

Spectral clustering is a method that first reduces the dimensions by representing data as an affinity matrix between samples and performs clustering by using the spectrum (eigenvalues) of the matrix.

Agglomerative clustering

Agglomerative clustering builds nested clusters by recursively merging the pair of clusters that minimally increases a given linkage distance.

Gaussian mixture

Gaussian mixture models are probabilistic models assuming that all the data are generated from a linear mixture of Gaussian distributions with unknown parameters and estimating their parameters by an Expectation-Maximization (EM) algorithm.

## Conclusion and future development

A database for heme proteins has been constructed to elucidate the structure–function relationships of heme and the origin of the diversity of the heme functions, leading to the functional predictions of unknown heme proteins and the *de novo* design of protein functions. The information for more than 13 000 hemes extracted from the PDB is stored in PyDISH with their axial ligands, information about the protein domain including them (Uniprot ID, protein fold by CATH ID, EC number, organism, molecular description, coordination distance), the orientation of the propionate sidechains, the protein function (structural keyword), the resolution of the structural data and the distortion of the heme porphyrin. Users can not only browse and download the data but also analyze heme porphyrin structures, by using the four analytical tools implemented in PyDISH (NSD, PCA, LDA and CAN). The PyDISH results can be utilized for designing of protein functions.

 For future developments, we will provide a more flexible data search in Browser. Auto-update of the database will be implemented in the near future to keep PyDISH up-to-date with PDB data. Furthermore, we will link each PyDISH entry and Gene Ontology terms for more exact description of protein function, attempt to globally analyze protein environment and electronic state of each heme in heme proteins and store the results in PyDISH. These data will be valuable to clarify the structure–function relationships in heme proteins.
